# Early Bone Infarction With Soft-tissue Abscess

**DOI:** 10.5435/JAAOSGlobal-D-21-00235

**Published:** 2022-07-15

**Authors:** Dongfang Ning, Feng Xu, Zhongxing Zhang, Xiaolong Yang, Jun Wei

**Affiliations:** From the Department of Bone and Joint Surgery, Liuzhou Municipal Liutie Central Hospital, Liuzhou, Guangxi, China.

## Abstract

Early bone infarction was rarely reported, and additional research is needed for diagnosis and treatment. This article aims to report the diagnosis and treatment process of a patient with unexplained early bone infarction with soft-tissue abscess and a review of related literature.

A 52-year-old male patient with early bone infarction of unknown etiology and soft-tissue abscess was analyzed retrospectively. He sustained right thigh swelling and pain for 5 days and hip and knee joint dysfunction, accompanied by fever, temperature 38°C, no limb numbness, sensory disturbance, and other discomfort. On MR images, early bone infarction with local soft-tissue abscess was found. After incision and drainage of soft-tissue abscess, wound débridement, and suture, the swelling and pain of the affected limb dysfunction gradually recovered.

Early bone infarction of unknown etiology with soft-tissue abscess has a low incidence. MRI is the main imaging diagnostic method. Conservative treatment is the main guard against malignant changes and surgical treatment, if necessary.

Bone infarction is the cellular death of bone components caused by the interruption of blood supply. It is a pathological state of bones that mostly occurs in the metaphysis or metaphyseal-diaphyseal area of the long bone, such as distal femur, proximal tibia, proximal fibula, and proximal humerus,^1^ and also occurs in axial bones (spine, sternum, ribs, and skull).^2,3^ Caisson disease, glucocorticoid, alcohol abuse, and dyslipidemia are considered as risk factors of bone infarction, and there are also case reports of special causes.^4^ Late bone infarction is clinically asymptomatic and relatively easy to diagnose. However, early bone infarction is manifested as the acute onset of severe pain and fever, which simulates infectious diseases, such as septic arthritis and acute osteomyelitis.^5^ It is easy to cause misdiagnosis in clinical work, prolong the hospital stay, and increase the cost. Early bone infarction was rarely reported, and additional research is needed for diagnosis and treatment. This article aims to report the diagnosis and treatment process of a patient with unexplained early bone infarction with soft-tissue abscess and a review of related literature.

## Case Presentation

**Figure 1:**
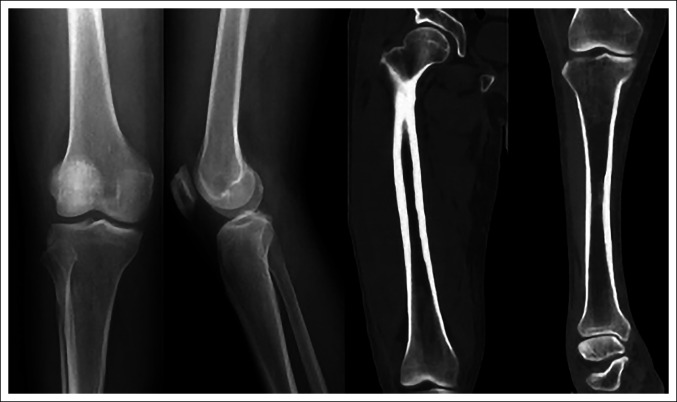
Radiograph and CT showing no obvious abnormalities in lower extremity.

**Figure 2:**
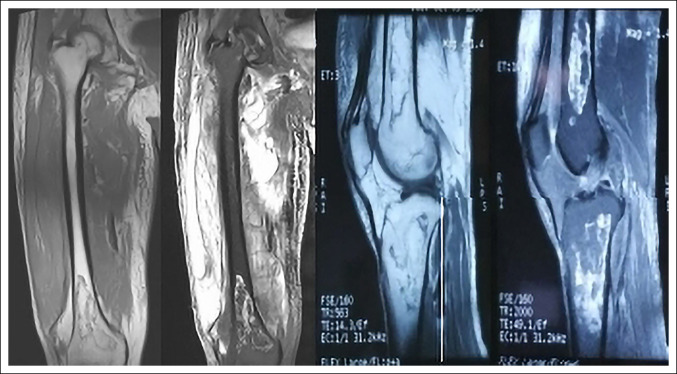
MRI showing early bone infarction in the distal femur and proximal tibia with local soft-tissue abscess in the right thigh, high signal on T1WI, and mixed signal on T2WI.

A 52-year-old male patient reported of redness, swelling, and pain in the middle and lower segments of the right thigh 5 days ago without obvious incentives, manifested as persistent swelling and pain. The pain could be relieved when lying on bed, accompanied by active flexion and extension dysfunction of the right hip and knee joint, fever, temperature 38°C; no limb numbness and sensory disorders; no limb joint stiffness and joint deformity; and no cough, expectoration, weight loss, night sweats, afternoon hot flashes, and other discomforts; symptoms gradually worsened, and he went to a local hospital for treatment, such as anti-infection, pain relief, and swelling. The specific process was unknown, but the symptoms had not improved. Therefore, he was transferred to our hospital for additional treatment. Since the onset of the disease, the spirit, appetite, and sleep had been normal; the urine and feces had been normal; and there had been no notable change in weight. No history of osteomyelitis, tumor, tuberculosis, arthritis, rheumatism, and immune disease was observed. The patient had no history of smoking and alcohol, hormones, immunosuppressants and other drug treatments, trauma, dyslipidemia, special anemic diseases (thalassemia, sickle cell anemia, etc), diving operations, and no relevant past medical interventions. Physical examination revealed no deformity of the right lower limb, and the skin was intact. The middle and lower segments of the right thigh were swollen, with obvious local tenderness, and the skin temperature was high, with no obvious fluctuation. From the supine position, the active range of motion of the right hip joint was observed as follows: flexion 0° to 30°, adduction 0° to 5°, abduction 0° to 10°, internal rotation 0° to 5°, and external rotation 0° to 10°; and the active range of motion of the right knee joint was 0° to 30°. The passive range of motion of the right hip joint was observed as follows: flexion 0° to 60°, adduction 0° to 15°, abduction 0° to 25°, internal rotation 0° to 15°, and external rotation 0° to 20°; and the passive range of motion of the right knee joint was 0° to 50°. The skin of the right lower limb was good, and the dorsal artery pulsation was normal. On hematology examination, the following results were found: white blood cells 3.85 × 10^9^/L, hemoglobin 95 g/L, platelets 185 × 10^9^/L, neutral ratio 93.6%, erythrocyte sedimentation rate 120 mm/hr, C-reactive protein 293.16 mg/L, normal blood lipid, and albumin 24 g/L. Immunohistochemistry was performed: interferon-gamma release assay for Mycobacterium tuberculosis (−), urine Ben-Zhou protein (−), immunofixation electrophoresis (−), autoimmune antibodies (−), tuberculosis antibody (−). No obvious abnormalities were found in lower extremity radiograph and CT (Figure 1). MRI showed early bone infarction in the distal femur and proximal tibia with local soft-tissue abscess in the right thigh (Figure 2). The soft-tissue abscess was punctured to extract coffee-like liquid, and multiple microbiological smears and microbial cultures of the abscess and blood were performed. No obvious bacterial and fungal growth was observed, and acid-fast staining of the pus (auramine ‟O” staining) and tuberculosis culture were negative. Bone marrow aspiration biopsy showed active bone marrow hyperplasia, markedly active granulation hyperplasia, normal erythroid hyperplasia, active macrophyte hyperplasia, scattered platelets, or small clusters. The soft-tissue abscess of the affected limb was treated with incision and drainage of the soft-tissue abscess by B-ultrasound and with empirical anti-infection, analgesic, nourishing support, etc. Three weeks after surgery, the swelling of the thigh subsided and the pain improved. After the tissue of the wound was fresh, the wound débridement and suture were done again. The dressing was changed after the operation, and the affected limb recovered 10 days later. The patient was discharged at 10 days postoperatively, and the preoperative symptoms almost disappeared. At the 1-month follow-up after discharge, the patient had good limb function and satisfactory results.

## Discussion

Caisson disease, glucocorticoid, alcohol abuse, dyslipidemia, trauma, and chemotherapy were considered as risk factors of bone infarction.^6^ In addition, bone infarction was usually found in special diseases, such as thalassemia, sickle cell disease, systemic lupus erythematosus, etc.^7,8^ Studies had shown that the pathogenesis of bone infarction stems from local blood supply disorders in the medullary cavity or bone cortex and acute vascular obstruction (thrombosis, lipid emboli, and fat cell hypertrophy with the compression of intraosseous capillaries) leads to ischemic osteonecrosis and bone infarction.^8-10^ In this study, the patient presented with early bone infarction of unknown etiology. There were no risk factors, including Caisson disease, glucocorticoid, alcohol abuse, dyslipidemia, trauma, chemotherapy, smoking and drinking, and other related medical history. On hematology examination, hemoglobin was 95 g/L. The bone marrow puncture result showed no blood system disease, and the hematologist considered it to be nutritional anemia. Kanthawang et al^8^ reported a case of early bone infarction in a patient with thalassemia undergoing splenectomy. However, this patient had nutritional anemia, no history of thalassemia and splenectomy, and mild anemia. Whether it is the cause of early bone infarction has not been confirmed.

Bone infarction has usually been asymptomatic and detected radiologically. Some patients have local pain in the lesion, mostly dull pain or swelling pain, no abnormal signs in the early stage, and local tenderness and restricted movement of adjacent joints in the late stage. Clinically, early bone infarcts may be accompanied by localized tenderness, swelling, erythema, fever, and leukocytosis,^5^ which simulates infectious diseases, such as septic arthritis and acute osteomyelitis. This patient had fever, redness, swelling, and pain in the affected limb; high local skin temperature; impaired mobility of adjacent joints; and high blood test indicators (erythrocyte sedimentation rate and C-reactive protein), which were in line with the clinical manifestations of early bone infarction.

On imaging, i.e., radiographs and CT, bone infarction in the early phases often has no obvious abnormalities or only shows bone density reduction. In the late phase, it is manifested by bone resorption and heterogeneous sclerosis in the infarcted area of the metaphyseal medullary cavity, surrounded by calcification or ossification, showing a ‟map-like” distribution of round or patchy high-density areas.^9^ Lafforgue thought that the periosteal reaction is the most common bone infarction and may be the only earliest radiograph sign,^1^ but few people realized that this sign may mean bone infarction and some differential diagnosis should be considered. Isolated periosteal reactions need to be evaluated for tumors or infections, such as chronic osteomyelitis and endogenous osteochondroma. MRI plays an important role in diagnosing bone infarction, especially in the diagnosis of early bone infarction, which had high sensitivity and specificity, and it is considered a more reliable imaging method for bone infarction.^8^ The early-phase MRI of bone infarction is characterized by medium or high signal on T1WI and high or mixed signal on T2WI, reflecting the bleeding or edema caused by the infarct focus, and the fat suppression sequence that is not enhanced by MRI reduces the high signal intensity of fat in the bone marrow and soft tissue. This highlights the pathological changes and helps improve the accuracy of diagnosis. On T1WI, the characteristic high signal intensity of bone, subperiosteal, and surrounding soft tissues is a powerful indicator of acute bone infarction.^8^ In the late phase, the dead bones show low signal on T1 and T2WI; new blood vessels and the surrounding granulation tissues show low signal on T1WI and high signal on T2WI, thus forming a characteristic change centered on the infarct. Map-like lesions are typical MRI manifestations of bone infarction.^1,3^ Acute osteomyelitis and early bone infarction have the same clinical manifestations of acute inflammation. It is often difficult to distinguish between the two through clinical and radiological manifestations (radiograph and CT), but they have different MRI manifestations. Early bone infarction shows high signal on T1WI and T2WI, subperiosteal and soft-tissue T1-high signal fluid, and no or very little signal enhancement. Acute osteomyelitis shows low-to-moderate signal on T1WI, high signal on T2WI, and subperiosteal and soft-tissue signal enhancement accompanied by abscess formation. Enhanced MR images usually show strong enhancement around the lesion. In osteomyelitis, there is a thick irregular enhancement ring, and the result of acute infarction is thin line edge enhancement.^5^ In nuclear medicine, in bone emission computed tomography and single-photon emission computed tomography/CT examinations, the imaging agent was concentrated in the bone infarction lesions, indicating that the metabolism is active here.^11-13^ However, the usefulness of nuclear medicine in diagnosing bone infarction has not been specifically evaluated. The main contribution of this research is that it can find other bone lesions.^1^ Bone infarction is a pathological process in which the blood supply of bone cells and bone marrow cells is interrupted, necrosis occurs, and the adipose bone marrow undergoes colloid transformation, cystic transformation, or liquefaction.^14^ From the pathological point of view, bone infarction can be divided into four areas: the central area of cell death, the ischemic injury area, the active hyperemia area, and the normal tissue area; the central area of cell death is surrounded by the ischemic injury area.^10^ Although pathological diagnosis is the benchmark for the diagnosis of bone infarction, if the imaging manifestations of bone infarction, especially MRI signs, are mastered, bone infarction can often be clearly diagnosed, and histopathological examination is generally not required for additional diagnosis.

Conservative treatment is the first choice for patients with asymptomatic and unobvious bone infarction. The treatment drugs include diphosphonates, hyperbaric oxygen, coenzyme Q10, erythropoietin, antihyperlipidemia, anticoagulants, antioxidants, and tissue repair protein, etc, but no particularly effective drugs have been found yet. Patients with unsatisfactory results of conservative treatment and additional development of bone infarction can choose surgical treatment according to the specific situation, such as drilling decompression, curettage and bone grafting, and joint replacement. However, there were also reports of osteosarcoma and fibrous histiocytoma on the basis of bone infarction.^15^ Therefore, the treatment of bone infarction should be paid attention to, with regular follow-up visits. When there is a tendency for malignancy, early surgical intervention is required.

In conclusion, in clinical work, bone infarction is relatively rare, especially early bone infarction. On radiographs, bone infarction lesions are often hidden and may have no clinical symptoms, which can easily cause misdiagnosis. MRI is the most effective way to diagnose bone infarction. MRI and other imaging methods can be used to understand the condition of bones and joints and to find bone infarction as soon as possible. Conservative treatment is the first choice for bone infarction, and surgical intervention can be used when the symptoms are severe or conservative treatment fails. In the process of treatment, we should be alert to the possibility of bone infarction malignant change, and additional research is needed for specific drugs.
